# Consequences of increasing convection onto patient care and protein removal in hemodialysis

**DOI:** 10.1371/journal.pone.0171179

**Published:** 2017-02-06

**Authors:** Nathalie Gayrard, Alain Ficheux, Flore Duranton, Caroline Guzman, Ilan Szwarc, Fernando Vetromile, Chantal Cazevieille, Philippe Brunet, Marie-Françoise Servel, Àngel Argilés, Moglie Le Quintrec

**Affiliations:** 1 RD–Néphrologie and EA7288, University of Montpellier, Montpellier, France; 2 Centre de dialyse Néphrologie Dialyse St Guilhem, Sète, France; 3 CoMET, INM Montpellier, NRI facilities, Montpellier, France; 4 Service de Néphrologie, Hôpital de La Conception–Université Aix-Marseille, Marseille, France; 5 European Uraemic Toxin Working Group of ESAO, endorsed by ERA-EDTA (EUTox), Krems, Austria; 6 Service de Néphrologie et Transplantation, Hôpital Lapeyronie CHU Montpellier, Montpellier, France; University of Glasgow, UNITED KINGDOM

## Abstract

**Introduction:**

Recent randomised controlled trials suggest that on-line hemodiafiltration (OL-HDF) improves survival, provided that it reaches high convective volumes. However, there is scant information on the feasibility and the consequences of modifying convection volumes in clinics.

**Methods:**

Twelve stable dialysis patients were treated with high-flux 1.8 m^2^ polysulphone dialyzers and 4 levels of convection flows (Q_UF_) based on _G_K_D-UF_ monitoring of the system, for 1 week each. The consequences on dialysis delivery (transmembrane pressure (TMP), number of alarms, % of achieved prescribed convection) and efficacy (mass removal of low and high molecular weight compounds) were analysed.

**Results:**

TMP increased exponentially with Q_UF_ (p<0.001 for N >56,000 monitoring values). Beyond 21 L/session, this resulted into frequent TMP alarms requiring nursing staff interventions (mean ± SEM: 10.3 ± 2.2 alarms per session, p<0.001 compared to lower convection volumes). Optimal convection volumes as assessed by _G_K_D-UF_-max were 20.6 ± 0.4 L/session, whilst 4 supplementary litres were obtained in the maximum situation (24.5 ± 0.6 L/session) but the proportion of sessions achieving the prescribed convection volume decreased from 94% to only 33% (p<0.001). Convection increased high molecular weight compound removal and shifted the membrane cut-off towards the higher molecular weight range.

**Conclusions:**

Reaching high convection volumes as recommended by the recent RCTs (> 20L) is feasible by setting an HDF system at its optimal conditions based upon the _G_K_D-UF_ monitoring. Prescribing higher convection volumes resulted in instability of the system, provoked alarms, was bothersome for the nursing staff and the patients, rarely achieved the prescribed convection volumes and increased removal of high molecular weight compounds, notably albumin.

## Introduction

Adding convection to standard haemodialysis was proposed in the sixties (haemofiltration) and seventies (hemodiafiltration (HDF)), to improve treatment performances [1,2]. Although the value of using convective over diffusive techniques has been debated for many years [3]. Three recent randomised controlled studies suggest a survival benefit associated with HDF, particularly when total convection volumes were high [4–6]. The Turkish study compared high flux haemodialysis with on-line HDF [4] while the Dutch CONTRAST [5] compared low flux haemodialysis with on-line HDF treated patients. Both studies only showed a survival benefit in post-hoc analyses that were not pre-specified (casting some doubt on their robustness). The Catalan ESHOL [6] only retained for analysis those patients with high convection volumes as per study design and observed a significant improvement in survival in HDF treated group. These reports may have participated in the wider use of HDF observed worldwide: OL-HDF, previously limited to 6.2% of the total hemodialysis has started increasing in Europe, and represents 10% in Australia and New Zealand [7–9]. The number of patients receiving HDF worldwide doubled between 2004 and 2010 [8].

However, the consequences of increasing convection volumes on the physics of the system and on its performances in a clinical situation have not been fully documented. These questions are particularly relevant since high convection volumes are obtained by increasing the convection flow, which depends on the transmembrane pressure (TMP) of the dialysis system. We previously studied the ratio of ultrafiltation flow over TMP, which represents the in situ global hydraulic permeability coefficient of the whole in vivo dialysis system (_G_K_D-UF_), [10,11]. We found that the ratio Q_UF_ / TMP followed a parabolic function which vertex is the optimal Q_UF_ setting of the dialysis system, in terms of TMP; it is the maximum value of _G_K_D-UF_ that we call _G_K_D-UF_-max. While the reproducibility of the _G_K_D-UF_-max determinations has been previously reported [12], the consequences of using this optimal convection flow in clinics remains to be described. The present work was designed to assess the feasibility and safety of different levels of convection flows, including the optimal setting guided by the _G_K_D-UF_ approach and the maximal convection volume possible according to the guidelines [13].

## Methods

### Patients and study design

**Fig 1:**
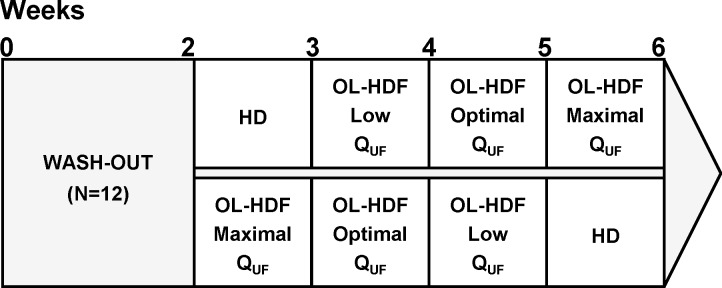
Schematic of the study protocol. The abbreviations are as follows: HD, hemodialysis; OL—HDF, On-line hemodiafiltration; Q_UF_, ultrafiltration flow.

Twelve stable dialysis patients were routinely treated in the dialysis clinic of Sète using HDF equipped dialysis monitors (Dialog+, B BRAUN, Melsungen, Germany) with alarms set following the recommendations of the European Renal Best Practice (ERBP) guidelines (ultrafiltration limited to 30% of the blood flow and TMP limited to 300 mmHg, as a safe maximum value) [13]. No automatic system adapting convection was used; the nursing staff had the prerogative of modifying the infusion flow if the TMP alarms prevented from pursuing the dialysis at the prescribed volumes. The patients were randomized into 2 groups, after 2 weeks of wash-out period with low flux dialysers. Six patients were successively allocated to standard high-flux haemodialysis treatment (one week) and to three post-dilutional OL-HDF treatments (one week each) with increasing convection volumes, including the maximum-possible (Fig 1). The six remnant patients had successive 1-week treatments with OL-HDF with decreasing convection volumes and ended with standard haemodialysis. In each sequence, patients were dialysed at the same total ultrafiltration flow (Q_UF_) for the complete week period. All patients were dialysed with high-flux 1.8 m^2^ polysulphone dialysers (Xevonta Hi 18, Amembris^®^ 1.8 m^2^, B Braun Avitum, Melsungen, Germany). All patients gave their written informed consent. The study protocol was approved by the ‘Comité de Protection des Personnes’ of Nîmes (2011.10.05 bis) with the registration number at the French Agency AFSSAPS 2011-A01092-39. The study was performed in agreement with the declaration of Helsinki.

### Dialysate and convection flow assessment

Total dialysate production flow was set at 600 mL/min and checked for every dialysis monitor (Table 2 shows the measurements). At the beginning of the first session of the week, _G_K_D-UF_-max was determined for every patient included in the study. To establish _G_K_D-UF_-max, infusion flow rate was set at 0 mL/min and then modified stepwise by 10 mL/min from 50 to 100 or 110 mL/min. After ~1 minute of stabilization, TMP was recorded and _G_K_D-UF_ calculated with QUF:

KG  D-UF = QUF × 60 / TMP

$$Q_{UF}\;=\;Q_{INF}+\;Q_{WL}$$

with _G_K_D-UF_, global ultrafiltration coefficient (mL.h^-1^.mmHg^-1^); Q_UF_, ultrafiltration flow (mL.min^-1^); TMP, transmembrane pressure (mmHg); Q_INF_, infusion flow (mL.min^-1^); Q_WL_, ultrafiltration flow for weight loss (mL.min^-1^).

The vertex of the parabolic tendency line (_G_K_D-UF_ over Q_UF_) is _G_K_D-UF-_max. The corresponding total convection flow is Q_UF_ at _G_K_D-UF_-max (X value of the _G_K_D-UF_-max point) which is considered the optimal convection OL-HDF. Low convection OL-HDF was defined as 60% of optimal and the maximum-possible Q_UF_ was aimed for respecting the limits advised by the ERBP.

### Dialysis achievement and alarms

All the data collected during dialysis sessions (time, pressures and flow rates for dialysate or blood, infusion flow, alarms and events) were recovered from the hard disk of each dialysis monitor and were processed on a spread-sheet (Excel, Microsoft). The clinical data (weight before and after dialysis, dialysis characteristics and events) were taken from the session sheets recorded by the nurses and physicians.

### Solute mass removal assessment

For every dialysis treatment, the total balance of selected substances was established using the continuous sampling of spent dialysate (CSSD)[14]. The concentration in the sampled spent dialysate of beta2-microglobulin (β2-m), retinol binding protein (RBP), alpha 1-anti-trypsin (α1AT), and albumin were determined by ELISA kits (ABCAM, Cambridge, UK). The concentration of lambda free light chains of immunoglobulins (λ FLC) was determined by ELISA kits (Bethyl laboratories, Montgomery, TX, USA) and the concentration of total proteins was determined by Bradford's method adapted for low concentration range [15]. The total mass removed of a given substance was obtained by multiplying the measured concentration by the total volume of dialysate.

Blood samples were obtained before and after dialysis of the mid-week treatment in order to assess dialysis efficacy (percentage reduction in urea and creatinine) as well as serum variation in haemoglobin, sodium, potassium, chloride, bicarbonate, calcium, phosphate, β2-m, albumin and total proteins.

### Adsorption studies and electron microscopy

For each convection condition, six dialysers were processed to assess protein adsorption to the membrane and morphology analysis by electron microscopy. The proteins adsorbed in the membrane of the dialyser were recovered following Mares *et al*’s method [16].

Two dialysers were mechanically cut after the EDTA–PBS rinses and ~1.5 cm length fibres were fixed with 2.5% glutaraldehyde in PHEM buffer, pH 7.2 for an hour at room temperature, followed by washing in PHEM buffer. Fixed samples were dehydrated using a graded ethanol series (30–100%), followed by 10 minutes in graded Ethanol—Hexamethyldisilazane. And then Hexamethyldisilazane alone. Subsequently, the samples were sputter coated with an approximate 10nm thick gold film and then examined under a scanning electron microscope (Hitachi S4000, at CoMET, MRI-RIO Imaging, Biocampus, INM Montpellier France) using a lens detector with an acceleration voltage of 10KV at calibrated magnifications.

## Statistics

Statistical analyses were performed using a SAS V9.2 (SAS Corporation, Cary, NC, USA). Differences in the continuous variables among the four different convection settings tested were assessed using an analysis of variance. Bonferroni’s test was used to check the differences between 2 of the 4 conditions. P values < 0.05 were considered significant. Values are given as mean ± standard error of the mean.

## Results

The clinical characteristics of the 12 patients (6 males and 6 females) included in the study are presented in Table 1. Table 2 displays the dialysis characteristics and the performances in terms of convection volume obtained with the different treatment types. The total convection volumes obtained in optimal OL-HDF were 20.6 ± 0.4 L/session and 24.5 ± 0.6 L/session with maximum OL-HDF. Increasing convection resulted in hemoconcentration within the blood circuit with a filtration fraction (QUF/Q_B_) of 3.2 ± 0.2, 14.8 ± 0.2, 23.9 ± 0.3 and 28.1 ± 0.4% (p<0.001) for HD, low, optimal and maximum convection OL-HDF respectively.

**Table 1:** Patients characteristics.

	Patients characteristics (N = 12)
**Gender**	6F / 6M
**Age (years)**	73 ± 12
**Body weight after (kg)**	71 ± 2
_**G**_**K**_**D-UF**_ **max (mL.h**^**-1**^**.mmHg**^**-1**^**)**	34.4 ± 1.2
**Haematocrit (%)**	35.5 ± 1.4
**Serum proteins (g.L**^**-1**^**)**	62.8 ± 1.2

**Table 2:** Convection flows and volumes as well as TMP values by dialysis condition.

Convection flow condition	HD	Low convection OL-HDF	Optimal convection OL-HDF	Maximum convection OL-HDF	p-values
**Dialysis characteristics**					
Session time (min)	232 ± 3	236 ± 3	235 ± 3	232 ± 3	0.24
Blood flow Q_B_ (mL.min^-1^)	365 ± 6	368 ± 5	364 ± 5	368 ± 5	0.41
Dialysate flow (mL.min^-1^)	602 ± 1	603 ± 1	602 ± 1	602 ± 1	0.69
UF flow for weight loss Q_WL_(mL.min^-1^)	11.9 ± 0.6	12.5 ± 0.5	12.3 ± 0.6	12.5 ± 0.5	0.73
Weight loss (kg)	2.8 ± 0.2	3.0 ± 0.1	2.9 ± 0.2	3.0 ± 0.1	0.71
Infusion flow Q_INF_ (mL.min^-1^)	0	41.7 ± 0.7	74.5 ± 1.0	90.9 ± 1.8	<0.001
Convection flow Q_UF_ = Q_WL_+ Q_INF_ (mL.min^-1^)	11.9 ± 0.7	54.1 ± 0.7	86.8 ± 1.1	103.5 ± 1.9	<0.001
Filtration fraction Q_UF_/Q_B_ (%)	3.2 ± 0.2	14.8 ± 0.2	23.9 ± 0.3	28.1 ± 0.4	<0.001
Infusion volume V_INF_ (L)	0	9.9 ± 0.2	17.7 ± 0.3	21.5 ± 0.5	<0.001
Convection volume V_UF_ (L)	2.8 ± 0.2	12.9 ± 0.2	20.6 ± 0.4	24.5 ± 0.6	<0.001
Mean TMP (mmHg)	79 ± 2	121 ± 2	185 ± 4	242 ± 4	<0.001
Max registered TMP (mmHg)	98 ± 2	152 ± 3	245 ± 7	322 ± 7	<0.001

### Influence of convection volume on dialysis safety/comfort and nursing work load

**Fig 2:**
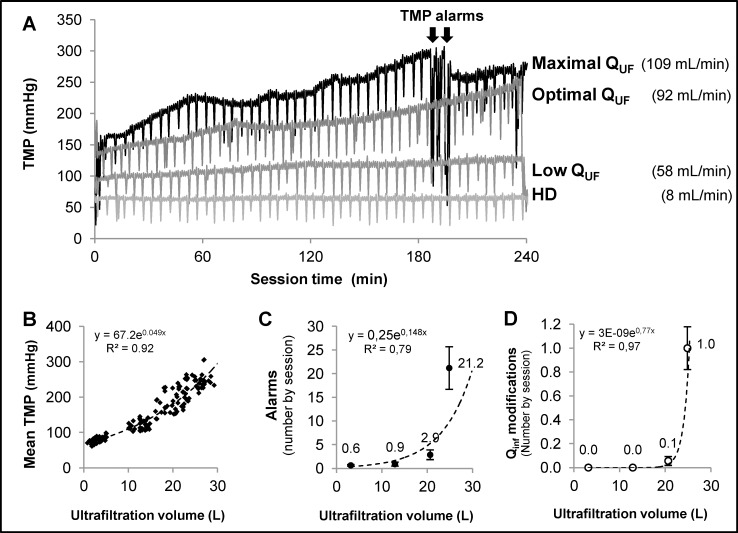
Panel **A) Example of TMP variations during dialysis**, for one patient with the four different convection volumes prescribed as to the protocol. TMP remained stable in haemodialysis and in low convection HDF while it increased during the treatment with optimal and maximum convection HDF. In the latter the increase was steeper and resulted in the appearance of TMP alarms that motivated nursing staff interventions. Panel B) depicts the **TMP over convection volume function**: each point represents the average of ~400 measurements during every dialysis of the 141 dialysis studied using a XEVONTA 1.8 m^2^, totaling >56,000 TMP measurements. Panel C) plots the **number of alarms** recorded during the assessed treatments and shows that it was around x10 fold in maximum when compared to optimal convection treatments. Panel D) plots the **number of reductions in infusion flow** necessary to avoid the alarms and pursue the treatment for the 4 treatment types, showing that infusion reduction was almost constantly required in maximum convection HDF at least in one occasion (a mean of 1 per session).

The total records of TMP during the complete dialysis session of a patient treated with the four different convection settings are given in Fig 2A as an example. It can be observed that TMP was stable in hemodialysis and low convection OL-HDF and increased in optimal OL-HDF and maximum convection OL-HDF which resulted in TMP alarms in the latter situation. The TMP recorded by the dialysis monitors of all the dialysis sessions are shown in Fig 2B, where it can be observed that TMP increased exponentially with convection flow.

A total of 920 alarms were recorded on 142 sessions (S1 Table). Treatment with maximum convection OL-HDF resulted in a mean of 21 alarms per session (Fig 2C), the majority of them being TMP alarms with an average of 8 minutes per session of accumulated time of by pass. The repeated TMP alarms frequently motivated a decrease in infusion flow by the nursing staff. Fig 2D illustrates the occurrence of convection flow reduction per dialysis session in the 4 different convection settings. It can be observed that the majority of the sessions performed with maximum convection OL-HDF required at least one manual intervention by the nurse decreasing the infusion flow. Some of the sessions required frequent nursing staff interventions. Consequently, the number of treatments achieving the prescribed convection volume fell from 94% to 33% when increasing from optimal to maximum convection OL-HDF (Table 3). Finally, Table 3 shows that 83% of the treatments with maximum convection had TMP alarms and 75% required extra-schedule nursing staff interventions, versus 9 and 6% in optimal OL-HDF and negligible for low convection OL-HDF and HD.

**Table 3:** Dialysis feasibility: Alarms, nursing staff interventions and convection modifications.

Convection flow condition	HD	Low convection OL-HDF	Optimal convection OL-HDF	Maximum convection OL-HDF	p-values
**Alarms**					
Sessions with TMP alarms (%)	0%	0%	9%	83%	<0.001
Mean non dalysis time due to alarms (min)	0.2 ± 0.1	0.3 ± 0.1	0.7 ± 0.3	8.0 ± 2.9	<0.001
**Nurse interventions to reduce infusion flow**					
Nurse interventions by sessions (%)	0%	0%	6%	75%	<0.001
Mean number of nurse interventions (nb/session)	0 ± 0	0 ± 0	0.06 ± 0.04	1.0 ± 0.13	<0.001
**Convection volume achievement**					
Sessions achieving the prescribed convection volume (%)	100%	100%	94%	33%	<0.001

### Influence of convection volume on efficacy

**Fig 3:**
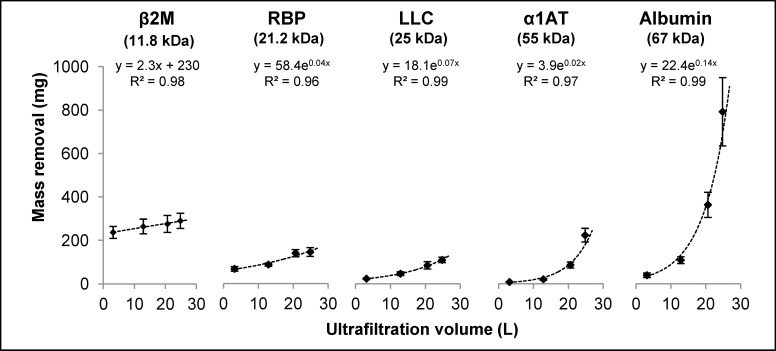
Removal of five different protein compounds in mg/session, analysed following the convection volumes obtained in the different dialysis conditions. It is observed that the variation in β-2m removal was not significantly different with HDF (slight linear increase with increasing convection volume), while the increase in removal was more evident in the higher mol wt range, being clearly exponential for albumin; albumin loss was around double when increasing from optimal convection to maximum convection HDF.

Removal of urea, creatinine, acid uric and phosphate are presented in Table 4. It can be seen that there was no significant difference in removal of these compounds when comparing dialysis with increasing convection volumes OL-HDF. Total protein removal was ~1200 mg and ~2300 mg per session in HD and maximum convection OL-HDF respectively. The total amount of β2-m, RBP, λ FLC, α1AT and albumin removed during the dialysis procedures and their variation according to the convection conditions are plotted in Fig 3. It can be observed that there was no significant difference in β2-m removal in the 4 different convection settings despite a small linear increase associated to the increase in convection. For the other compounds, the higher the molecular weight (mol wt) the more pronounced the increase in mass removal with convection volume. In the case of albumin, the removed mass increased 20-fold when comparing maximum convection OL-HDF (with 793 ± 158 mg/session) to dialysis (39 ± 10 mg/session).

**Table 4:** Dialysis efficacy.

Convection flow condition	HD			Low convection OL-HDF			Optimum convection OL-HDF			Maximum convection OL-HDF			p-values
**Urea**													
Blood before (mmol/L)	17,0	±	1,6	17,5	±	1,8	19,3	±	2,1	17,4	±	1,6	0,15
Blood after (mmol/L)	3,9	±	0,5	3,5	±	0,5	3,5	±	0,6	3,6	±	0,5	0,22
Blood RR (%)	80%	±	1%	81%	±	1%	81%	±	1%	80%	±	1%	0,52
Total mass* (mmol)	526	±	38	508	±	37	545	±	43	473	±	32	0,21
Clearance (mL/min)	228	±	10	231	±	7	227	±	8	239	±	8	0,24
**Creatinine**													
Blood before (μmol/L)	616	±	44	607	±	48	628	±	46	616	±	41	0,05
Blood after (μmol/L)	180	±	23	165	±	21	163	±	22	175	±	19	0,10
Blood RR (%)	75%	±	1%	74%	±	2%	74%	±	2%	73%	±	2%	0,93
Total mass* (μmol)	12184	±	965	11855	±	847	12268	±	904	11701	±	917	0,51
Clearance (mL/min)	138	±	7	138	±	8	135	±	8	140	±	8	0,41
**Uric acid**													
Blood before (μmol/L)	285	±	19	291	±	14	318	±	19	283	±	16	0,02
Blood after (μmol/L)	59	±	6	52	±	4	56	±	6	53	±	5	0,36
Blood RR (%)	81%	±	2%	82%	±	1%	83%	±	1%	82%	±	1%	0,53
Total mass* (μmol)	6753	±	319	6757	±	273	7209	±	338	6735	±	347	0,33
Clearance (mL/min)	193	±	12	193	±	7	196	±	13	202	±	10	0,76
**Phosphorus**													
Blood before (mmol/L)	1,35	±	0,15	1,32	±	0,11	1,32	±	0,11	1,23	±	0,09	0,10
Blood after (mmol/L)	0,51	±	0,05	0,51	±	0,04	0,51	±	0,04	0,48	±	0,04	0,49
Blood RR (%)	64%	±	3%	59%	±	3%	59%	±	4%	58%	±	4%	0,25
Total mass * (μmol)	888	±	86	900	±	105	942	±	89	831	±	59	0,15
Clearance (mL/min)	144	±	13	145	±	11	161	±	13	150	±	13	0,22
**Total protein**													
Blood before (g/L)	63	±	2	60	±	2	60	±	2	62	±	2	0,08
Blood after (g/L)	57	±	2	56	±	2	54	±	2	57	±	2	0,46
Blood RR (%)	10%	±	2%	7%	±	2%	9%	±	2%	8%	±	3%	0,80
Total mass * (mg)	1204	±	79	1438	±	79	1882	±	113	2329	±	118	<0.001
Clearance (mL/min)	0,077	±	0,005	0,10	±	0,01	0,13	±	0,01	0,16	±	0,01	<0.001
**β2M**													
Blood before (mg/L)	31,43	±	1,94	31,57	±	1,99	29,54	±	2,07	30,94	±	1,66	0,48
Blood after cor. (mg/L)	9,61	±	1,04	6,90	±	0,59	5,33	±	0,76	5,94	±	0,67	<0.001
Blood RR (%)	74%	±	3%	82%	±	2%	85%	±	2%	84%	±	2%	<0.001
Total mass* (mg)	237	±	27	260	±	31	274	±	35	290	±	35	0,26
Clearance (mL/min)	56	±	5	75	±	11	83	±	12	88	±	11	0,02
**Albumin**													
Blood before (g/L)	32,13	±	1,44	31,85	±	1,27	32,14	±	1,41	33,65	±	0,51	0,31
Blood after cor.(g/L)	28,43	±	0,66	27,52	±	1,68	27,65	±	1,26	28,75	±	1,14	0,88
Blood RR (%)	10%	±	4%	13%	±	4%	14%	±	2%	15%	±	3%	0,87
Total mass* (mg)	39	±	10	116	±	16	386	±	57	793	±	158	<0.001
Clearance (mL/min)	0,006	±	0,002	0,014	±	0,002	0,045	±	0,008	0,084	±	0,007	<0.001

*Total mass removed in dialysate by session

To illustrate the variation of removal with increasing convection according to protein size, β2-m and albumin were compared. With conventional dialysis, six times more β2-m than albumin was removed (ratio of removed albumin / removed β2-m = 0.167). The ratio was reversed by increasing convection and the total amount of removed albumin was 3-fold that of removed β2-m, with maximum convection OL-HDF (ratio = 2.96). The albumin / β2-m removal ratio increased by 14.3 fold when passing from dialysis to the maximum convection OL-HDF situation (S1 Fig).

**Fig 4:**
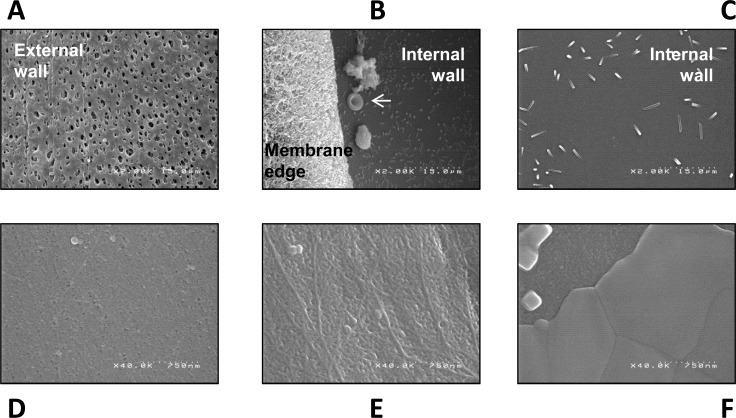
Electron microscopy of the membrane AMEMBRIS used in the study. An example of one dialyser used in hemodialysis is given in the upper panels (A, B and C; x2,000 magnification) and of one dialyser used in hemodiafiltration in the lower panels (D, E and F; x40,000 magnification). (A) The external wall displays visible pores, significantly larger than the pores of the internal wall (B and C), which are not visible at x2,000 magnification. (B) The arrow points at one erythrocyte located between 2 cumulated material and crystals from the rinsing fluid (saline and sodium dodecyl sulphate). (C) Sparse crystals are laying on the internal wall. Three different zones of the internal wall of the dialyser are displayed in the lower panels at x40,000 magnification, one with practically all the pores visible and accessible (D), one with some pores covered by a proteinaceous material (E) and one where all the pores are covered by a uniform material attached to the membrane (F).

Electron microscopy of AMEMBRIS membranes after treatment clearly characterised a change in membrane pores after cake formation during the dialysis procedure (Fig 4). The total amount of adsorbed proteins was rather small (41 ± 27, 88 ± 34, 74 ± 33 and 56 ± 30 mg/session respectively in haemodialysis, low, optimal and maximal OL-HDF), and it was not associated with convection. The total amount of adsorbed proteins in dialyser membranes was between 2.4 and 6.1% of the amount of proteins eliminated through the dialysate.

## Discussion

Convection was sought from early sixties trying to establish a renal replacement system based upon a filtration process rather than upon a diffusion process, aiming to emulate the filtration process of the glomerulus in the “in vivo” situation [1,2]. The present study is the first controlled study designed to assess the effects of increasing convection in clinics and provides new data on the clinical feasibility and consequences of using high convection volumes in OL-HDF, a feature that the recent RCTs suggest is of crucial importance on seeking survival benefits in dialysis treatment [4–6].

The analysis of _G_K_D-UF_ allows identifying the optimal convection in terms of differential convection obtained by a differential TMP required in a dialysis system [11,12]. We found that OL-HDF performed with optimal convection obtained total convection volumes around 21 L/session, which is well within the range reported to be beneficial by the 3 RCTs analysing survival in dialysis patients [4–6]. We found that higher convection volumes were associated with an exponential increase in TMP. TMP in the latter situation frequently exceeded the upper programmed limits, resulting in alarms and staff interventions to decrease infusion pump speed. In consequence, increasing convection over the optimal convection flow was cumbersome both for patients and for the nursing staff (blood clotting, TMP alarms) and the final convection volumes rarely reached those initially prescribed.

To reduce the problems appearing when increasing convection in post-dilutional OL-HDF, various forms of HDF have been developed varying the point of infusion of the replacement fluid, referred to as pre-, mid- or mixed-dilution. Although these methods allow higher convection flow with less alarms, their efficiency in small mol wt compound clearances is recognized to be lower than that obtained in post-dilution and therefore they deserve further studies before being proposed indiscriminately [17–20].

Our data show that removal of small soluble compounds is not significantly enhanced by convection, while differences are visible on higher molecular weight uraemic retention solutes from the middle molecule group [21,22]. We have previously observed in studies analyzing the instantaneous clearances [18] an increase in small molecule clearance proportional to the convection in post-dilution HDF and a decrease in clearance also proportional to convection in pre-dilutional HDF and we explained these variations mainly by the modifications of the concentration at the blood inlet and consequently in blood to dialysate side gradient.In the present study, however, assessing the complete dialysis session, we did not observe a significant increase in total removal or in percentage reduction of serum levels proportional to convection. Although surprising, these results are in accordance with other reports [23]. Higher removal in high convection HDF may result in a steeper decrease in serum level during the dialysis session, thereby decreasing faster the concentration blood to dialysate gradient and consequently, removal by diffusion during the dialysis session in the higher convection situation, blunting the expected effect of a higher instant clearance on the total length of the dialysis treatment. This hypothesis needs to be checked by further studies, probably monitoring serum levels throughout the dialysis session, which are out of the main aims of the present work.

Concerning the middle molecules, increasing convection (and therefore, TMP) resulted in increasing the removal of high mol wt compounds, while the increase observed in β2-m (11.8 kDa mol wt) removal was not significant or relevant. It was clearly seen that conventional dialysis was highly selective in protein removal with a 6-fold greater removal of β2-m than albumin (67 kDa), whilst it is 1000-fold less abundant in serum (β2-m: 32±1 mg/L; albumin: 32100 ± 1400 mg/L). This selectivity diminished with increasing convection, and at the maximum convection OL-HDF, the ratio albumin / β2-m removal was reversed. Expanding the convection over the optimal setting resulted in increasing total protein removal by around 0.5 g/session (from 1.8 to 2.3 g/session) and of this 0.4 g (80%) consisted of albumin. These findings suggest that higher convection volumes and elicited TMP result in an upward shift of the molecular weight cut-off of the dialysis system, in keeping with the studies of Ahrenholz *et al* [24]. The existence of the shift in the cut-off of a dialysis system is very relevant in clinics: on the one hand, albumin is an unchallenged prognostic factor in uraemia [25,26] and on the other, some authors have proposed to remove it as a means to clear protein bound uraemic toxins [27]. Nevertheless, whether the albumin-associated removal of protein-bound uraemic toxins participates in the suggested survival benefit of high volume OL-HDF cannot be concluded based on presently available data. Finally, no clear influence of convection was observed on protein removal by adsorption, which had a minor impact on total removal, as it represented between 2,4 and 6,1% of the total protein removed.

Systems exist helping to achieve high convection volumes in post dilution OL-HDF. These systems are very efficient as they allow maximum filtration rates and consequently high convection volumes [28–30]. They have an automatic adaptation of convection which avoids TMP alarms whereas the modification of the infusion rates was manual by the nursing staff in our unit. The convection volumes achieved by these systems are very close to those we obtained in our maximum convection setting, however, attention has to be paid to albumin loss and to the fact that the automatic modifications of the infusion rate (and the convection volumes) may be overseen by the clinician.

In conclusion, optimal convection OL-HDF as defined by _G_K_D-UF_-max allows convection volumes (>20 L per session) within the range of those found to be associated with survival benefits by some recent RCTs [4–6] and increases removal, particularly of middle molecules. Trying to push convection above optimal OL-HDF results in a significant increase in removal of large mol wt compounds, and particularly albumin, showing that the cut-off of a dialysis system is not a constant, but instead depends on TMP and convection. Pushing convection above optimal is frequently associated with TMP-related alarms, necessitating supplementary control systems to improve clinical feasibility and reaching the prescribed volumes. The findings observed in this controlled group of patients are relevant and informative for the prescriber, the renal physician, as well as for the nursing staff taking care of the patient and ensuring the execution of the prescribed treatment. These results should help in improving the treatments that we offer to patients. Should we want to limit the convection volume to avoid its associated problems, alternative ways to improve dialysis may include increasing membrane surface, treatment time, and if possible, blood flows.

## Supporting information

S1 Fig Ratio of albumin removal to β2-m removal by ultafiltration volume. Mean and standard error of the mean for each convection condition.
(PDF)

S1 Table Description of the type and frequency of the recorded alarms. The p values of the ANOVA test are given in the right hand side column. All the variables that were significantly different when analysing the four groups, were also different when comparing the over GKD-UF-max to the others by X^2^ or Bonferroni analysis.
(DOCX)
