# Correction: Consequences of increasing convection onto patient care and protein removal in hemodialysis

**DOI:** 10.1371/journal.pone.0190761

**Published:** 2018-01-02

**Authors:** Nathalie Gayrard, Alain Ficheux, Flore Duranton, Caroline Guzman, Ilan Szwarc, Fernando Vetromile, Chantal Cazevieille, Philippe Brunet, Marie-Françoise Servel, Àngel Argilés, Moglie Le Quintrec

The Corresponding Author’s email address appears incorrectly. The correct email address is: argiles@rd-n.org.

There is an error in Table 4. The unit is incorrect for the line Total Mass under the heading Phosphorous in the first column. The unit should read: Total mass (mg). Please see the corrected Table 4 here.

**Table 4:** Dialysis efficacy.

Convection flow condition	HD			Low convection OL-HDF			Optimum convection OL-HDF			Maximum convection OL-HDF			p-values
**Urea**													
Blood before (mmol/L)	17.0	±	1.6	17.5	±	1.8	19.3	±	2.1	17.4	±	1.6	0.15
Blood after (mmol/L)	3.9	±	0.5	3.5	±	0.5	3.5	±	0.6	3.6	±	0.5	0.22
Blood RR (%)	80%	±	1%	81%	±	1%	81%	±	1%	80%	±	1%	0.52
Total mass* (mmol)	526	±	38	508	±	37	545	±	43	473	±	32	0.21
Clearance (mL/min)	228	±	10	231	±	7	227	±	8	239	±	8	0.24
**Creatinine**													
Blood before (μmol/L)	616	±	44	607	±	48	628	±	46	616	±	41	0.05
Blood after (μmol/L)	180	±	23	165	±	21	163	±	22	175	±	19	0.10
Blood RR (%)	75%	±	1%	74%	±	2%	74%	±	2%	73%	±	2%	0.93
Total mass* (μmol)	12184	±	965	11855	±	847	12268	±	904	11701	±	917	0.51
Clearance (mL/min)	138	±	7	138	±	8	135	±	8	140	±	8	0.41
**Uric acid**													
Blood before (μmol/L)	285	±	19	291	±	14	318	±	19	283	±	16	0.02
Blood after (μmol/L)	59	±	6	52	±	4	56	±	6	53	±	5	0.36
Blood RR (%)	81%	±	2%	82%	±	1%	83%	±	1%	82%	±	1%	0.53
Total mass* (μmol)	6753	±	319	6757	±	273	7209	±	338	6735	±	347	0.33
Clearance (mL/min)	193	±	12	193	±	7	196	±	13	202	±	10	0.76
**Phosphorus**													
Blood before (mmol/L)	1.35	±	0.15	1.32	±	0.11	1.32	±	0.11	1.23	±	0.09	0.10
Blood after (mmol/L)	0.51	±	0.05	0.51	±	0.04	0.51	±	0.04	0.48	±	0.04	0.49
Blood RR (%)	64%	±	3%	59%	±	3%	59%	±	4%	58%	±	4%	0.25
Total mass* (mg)	888	±	86	900	±	105	942	±	89	831	±	59	0.15
Clearance (mL/min)	144	±	13	145	±	11	161	±	13	150	±	13	0.22
**Total protein**													
Blood before (g/L)	63	±	2	60	±	2	60	±	2	62	±	2	0.08
Blood after (g/L)	57	±	2	56	±	2	54	±	2	57	±	2	0.46
Blood RR (%)	10%	±	2%	7%	±	2%	9%	±	2%	8%	±	3%	0.80
Total mass* (mg)	1204	±	79	1438	±	79	1882	±	113	2329	±	118	<0.001
Clearance (mL/min)	0.077	±	0.005	0.10	±	0.01	0.13	±	0.01	0.16	±	0.01	<0.001
**β2M**													
Blood before (mg/L)	31.43	±	1.94	31.57	±	1.99	29.54	±	2.07	30.94	±	1.66	0.48
Blood after cor. (mg/L)	9.61	±	1.04	6.90	±	0.59	5.33	±	0.76	5.94	±	0.67	<0.001
Blood RR (%)	74%	±	3%	82%	±	2%	85%	±	2%	84%	±	2%	<0.001
Total mass* (mg)	237	±	27	260	±	31	274	±	35	290	±	35	0.26
Clearance (mL/min)	56	±	5	75	±	11	83	±	12	88	±	11	0.02
**Albumin**													
Blood before (g/L)	32.13	±	1.44	31.85	±	1.27	32.14	±	1.41	33.65	±	0.51	0.31
Blood after cor.(g/L)	28.43	±	0.66	27.52	±	1.68	27.65	±	1.26	28.75	±	1.14	0.88
Blood RR (%)	10%	±	4%	13%	±	4%	14%	±	2%	15%	±	3%	0.87
Total mass* (mg)	39	±	10	116	±	16	386	±	57	793	±	158	<0.001
Clearance (mL/min)	0.006	±	0.002	0.014	±	0.002	0.045	±	0.008	0.084	±	0.007	<0.001

*Total mass removed in dialysate by session
