# Supplementary figures and images for: Consequences of increasing convection onto patient care and protein removal in hemodialysis
Source: PLoS One. 2017 Feb 6;12(2):e0171179. doi: 10.1371/journal.pone.0171179 (PMC5293266; doi:10.1371/journal.pone.0171179)

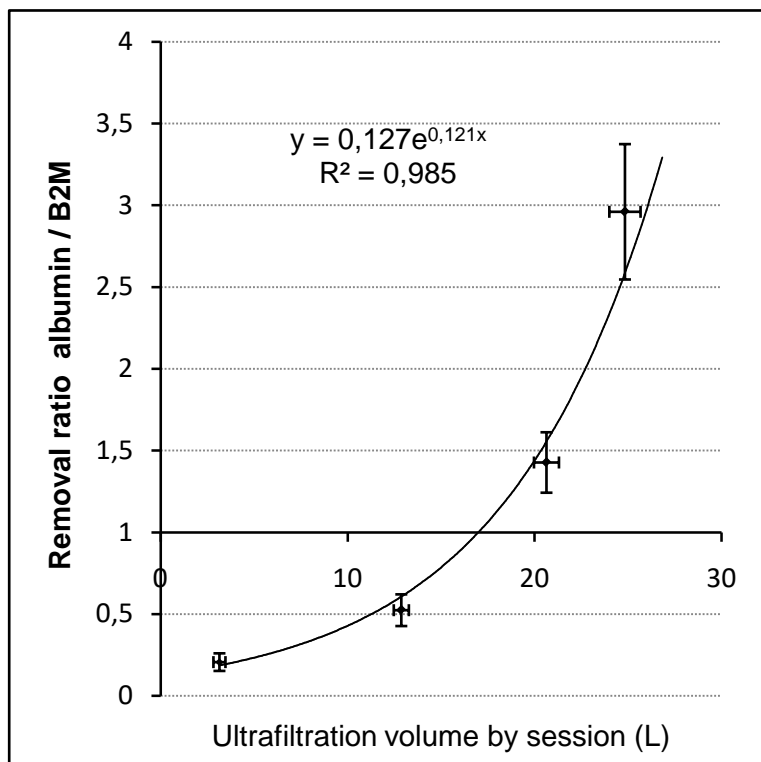

**S1 Fig**

Supplement: S1 Fig — Mean and standard error of the mean for each convection condition. (PDF) [file pone.0171179.s001.pdf]
