# Supplementary material for: Consequences of increasing convection onto patient care and protein removal in hemodialysis
Source: PLoS One. 2017 Feb 6;12(2):e0171179. doi: 10.1371/journal.pone.0171179 (PMC5293266; doi:10.1371/journal.pone.0171179)
Supplement: S1 Table — The p values of the ANOVA test are given in the right hand side column. All the variables that were significantly different when analysing the four groups, were also different when comparing the over GKD-UF-max to the others by X2 or Bonferroni analysis. (DOCX) [file pone.0171179.s002.docx]

**S1 table**. **Description of the type and frequency of the recorded alarms**. The p values of the ANOVA test are given in the right hand side column. All the variables that were significantly different when analysing the four groups, were also different when comparing the over _G_K_D-UF_-max to the others by X² or Bonferroni analysis.

|  | HD | Low convection OL-HDF | Optimal convection OL-HDF | Maximum convection OL-HDF | *p* |
| --- | --- | --- | --- | --- | --- |
| **Nb of sessions assessed** | 35 | 35 | 36 | 36 |  |
| **TMP out of alarm limits** |  |  |  |  |  |
| Nb sessions with alarms | 0 | 0 | 3 | 29 | <0.0001 |
| Total number of alarms | 0 | 0 | 41 | 372 | <0.0001 |
| Total number of alarms/nb of sessions assessed | 0 | 0 | 1.1 | 10.3 | <0.0001 |
| **Arterial pressure - lower limit** |  |  |  |  |  |
| Nb sessions with alarms | 2 | 3 | 3 | 14 | <0.0001 |
| Total number of alarms | 3 | 4 | 8 | 38 | 0.0006 |
| Total number of alarms/nb of sessions assessed | 0.1 | 0.1 | 0.2 | 1.1 | 0.0006 |
| **Arterial pressure - upper limit** |  |  |  |  |  |
| Nb sessions with alarms | 2 | 0 | 4 | 9 | 0.0047 |
| Total number of alarms | 2 | 0 | 5 | 14 | 0.0166 |
| Total number of alarms/nb of sessions assessed | 0.1 | 0 | 0.1 | 0.4 | 0.0166 |
| **Pressure at blood inlet -lower limit** |  |  |  |  |  |
| Nb sessions with alarms | 1 | 0 | 3 | 5 | 0.0792 |
| Total number of alarms | 1 | 0 | 3 | 7 | 0.0377 |
| Total number of alarms/nb of sessions assessed | 0 | 0 | 0.1 | 0.2 | 0.0377 |
| **Pressure at blood inlet -upper limit** |  |  |  |  |  |
| Nb sessions with alarms | 0 | 0 | 2 | 8 | 0.0004 |
| Total number of alarms | 0 | 0 | 6 | 34 | 0.0014 |
| Total number of alarms/nb of sessions assessed | 0 | 0 | 0.2 | 0.9 | 0.0014 |
| **Venous pressure - lower limit** |  |  |  |  |  |
| Nb sessions with alarms | 9 | 4 | 8 | 17 | 0.0065 |
| Total number of alarms | 11 | 4 | 15 | 102 | <0.0001 |
| Total number of alarms/nb of sessions assessed | 0 | 0 | 0.4 | 2.8 | <0.0001 |
| **Venous pressure - upper limit** |  |  |  |  |  |
| Nb sessions with alarms | 4 | 8 | 12 | 30 | <0.0001 |
| Total number of alarms | 5 | 24 | 25 | 196 | <0.0001 |
| Total number of alarms/nb of sessions assessed | 0.1 | 0.7 | 0.7 | 5.4 | <0.0001 |
| **Total number of alarms** |  |  |  |  |  |
| Total number of alarms | 22 | 32 | 103 | 763 | <0.0001 |
| Total number of alarms/nb of sessions assessed | 0.6 | 0.9 | 2.9 | 21.2 | <0.0001 |
| **Alarm-related nurse interventions to reduce infusion flow** |  |  |  |  |  |
| Nb sessions with interventions | 0 | 0 | 2 | 27 | <0.0001 |
| Total number of interventions | 0 | 0 | 2 | 36 | <0.0001 |
| Total number of interventions/nb of sessions assessed | 0 | 0 | 0.06 | 1.00 | <0.0001 |
| **Non-dialysis time due to alarms** |  |  |  |  |  |
| Nb sessions with non dialysis time | 12 | 4 | 12 | 29 | <0.0001 |
| Total non dialysis time in mn | 6 | 10 | 26 | 289 | <0.0001 |
| Total non dialysis time in mn/nb of sessions assessed | 0.2 | 0.3 | 0.7 | 8.0 | <0.0001 |
| **% of dialysis achieving the prescribed volume (V_presc_)** |  |  |  |  |  |
| Nb sessions achieving V_presc_ | 35 | 35 | 34 | 12 | <0.0001 |
| Nb sessions achieving V_presc_ /nb of sessions assessed in % | 100% | 100% | 94% | 33% | <0.0001 |
